# Lithium Squarate as Sacrificing *Electrolyte* Additive for Prelithiation: Case Study in Zero‐Excess Lithium Metal Batteries

**DOI:** 10.1002/advs.202517221

**Published:** 2025-12-14

**Authors:** Ibrahim Lawan Abdullahi, Anindityo Arifiadi, Alexandros Tsoufios, Nick Fehlings, Silvan Stuckenberg, Lukas Stolz, Dominik Voigt, Martin Winter, Johannes Kasnatscheew

**Affiliations:** ^1^ MEET Battery Research Center Institute of Physical Chemistry University of Münster Corrensstraße 46 48149 Münster Germany; ^2^ International Graduate School for Battery Chemistry Characterization Analysis Recycling and Application (BACCARA) University of Münster Corrensstraße. 40 48149 Münster Germany; ^3^ Helmholtz‐Institute Münster IMD‐4 Forschungszentrum Jülich GmbH Corrensstraße 46 48149 Münster Germany

**Keywords:** additive depletio, high energy anodes, oxidation onset, prelithiation, sacrificing additives

## Abstract

Among different approaches, the prelithiation via sacrificing additives can be technically relatively easy applied. In this work, lithium squarate (Li_2_C_4_O_4_) as a literature‐known representative is investigated in zero‐excess lithium metal batteries, which are simple in handling and have high active lithium loss (ALL), thus ideal for R&D of sacrificing additives. When incorporated via cathode, Li_2_C_4_O_4_ oxidizes at a relatively low cathode potential (≈4.5 V vs Li|Li⁺) and provides the aimed active Li (= capacity), but ruptures the cathode via gaseous evolution of CO and CO_2_. Interestingly, when incorporated via electrolyte, the additive oxidation is absent. This can be correlated with its reductive depletion in the course of solid electrolyte interphase (SEI) formation, as hinted via computational analysis by its relatively low energetic level of lowest unoccupied molecular orbital (LUMO), as well as by energy dispersive x‐ray spectroscopy, and decreased gas evolution. Hence, the squarate is concluded to be impractical as an additive in electrolytes when combined with anodes, which *in operando* form the SEI (e.g., graphite, Si). In Li metal cells, i.e., with an already existing and passivating “preformed” native SEI, the squarate oxidation can be seen again, but the oxidation onset sensitively depends on the cathode type and electrolyte formulation.

## Graphical abstract

Active lithium loss (ALL) and capacity fade can be compensated by prelithiation, apparently simple via sacrificing additives e.g., lithium squarates. However, as a cathode additive it ruptures the cathode via gas evolution, while as an electrolyte additive it gets reductively depleted on anode side in course of solid electrolyte interphase (SEI) formation.

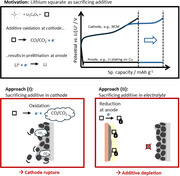

## 1 Introduction

Lithium‐ion batteries (LIBs) have emerged as a vital technology powering devices ranging from, e.g., smartphones^[^ ^1^ ^]^ to electric vehicles.^[^ ^2^ ^]^ However, in the course of increasing demands, the LIBs require further R&D.^[^ ^3^ ^]^ The conventional LIBs, consisting of graphite‐based anodes and lithium transition metal oxide‐based cathodes, i.e., LiNi*_x_* Co*_y_* Mn*_z_* O_2_ (*x*+*y*+*z* = 1; NCM), progressively reach the apex in practical specific energy.^[^ ^4^ ^]^

Exchanging graphite (372 mAh g^−1^) with Li metal as anode (3860 mAh g^−1^)^[^ ^5^ ^]^ to obtain so‐called Li‐metal batteries (LMBs) can boost specific energy.^[^ ^6^, ^7^ ^]^ However, they suffer from safety and cycle life issues in the course of formation of high surface area lithium (HSAL), e.g., lithium dendrites, leading to continuous formation of solid electrolyte interphase (SEI), dead lithium, and resistance rises.^[^ ^8^, ^9^, ^10^, ^11^, ^12^, ^13^ ^]^

**Figure 1:**
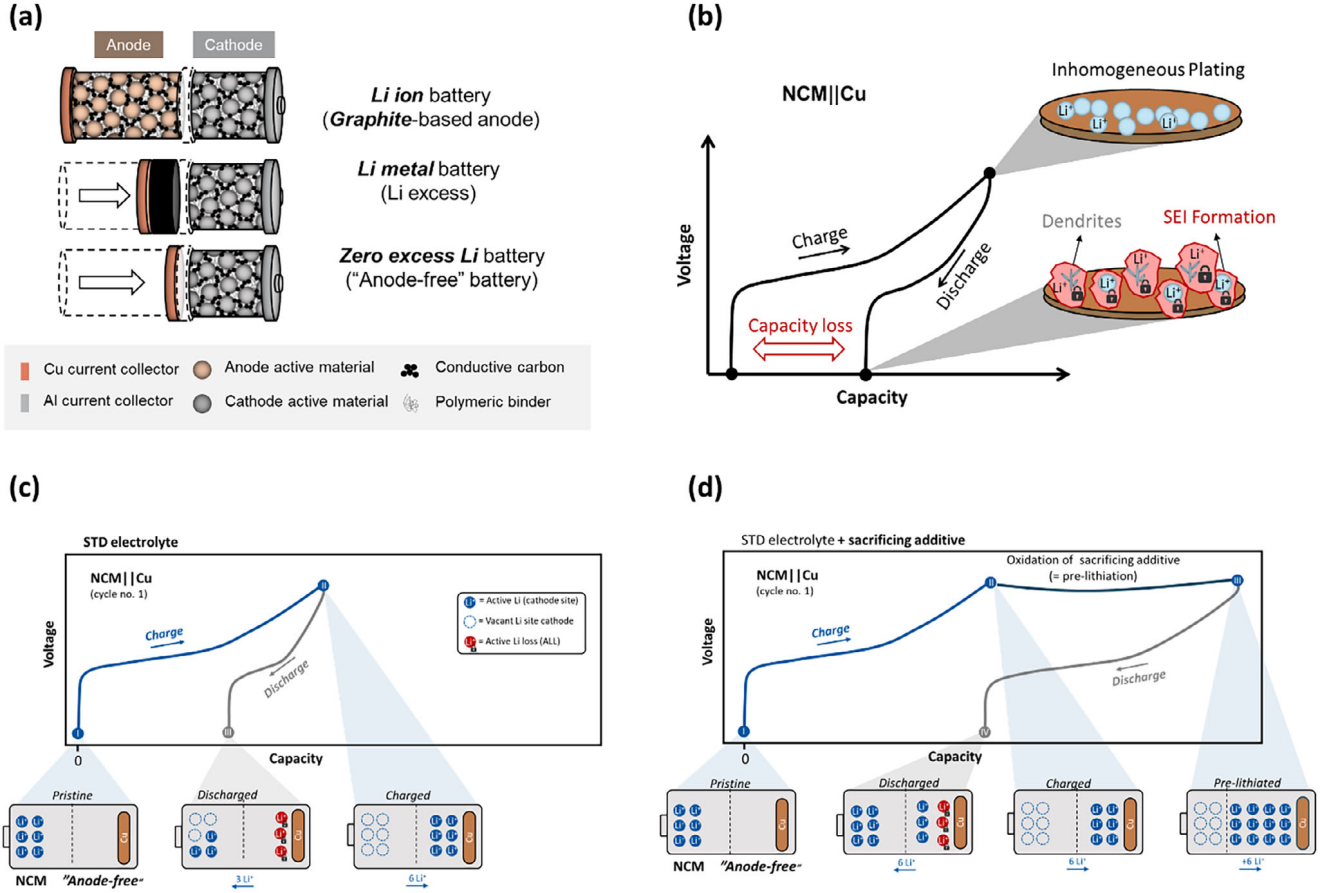
a) A schematic of LIB, LMB, and ZELMB in terms of decreased cell volume, respectively (redrawn from ref. [23]). b) Initial charge/discharge voltage profile showcasing the capacity loss due to inhomogeneous Li plating and accompanied by ALL in the course of HSAL, e.g., Li dendrites, and SEI formation. Redrawn from ref.[23] c) ALL decreases Li inventory and specific discharge capacity, resulting in incomplete re‐lithiated cathode. d) Pre‐lithiation via, e.g., sacrificing additives provides active Li in the course of additive oxidation, which compensates ALL and creates a Li reservoir, which enhances discharge capacity by a full re‐lithiation of the cathode.

Zero excess lithium metal batteries (ZELMBs) can be regarded as the “purest” LMB, as the cell is assembled without Li foil/Li excess, and the Li metal only emerges during plating in the course of charge. As the Cu current collector is bare in a pristine state, i.e., no active/inactive materials, they offer the highest possible gravimetric/volumetric energy for anodes (**Figure** **1**a). Here, the lithium inventory is solely provided by the cathode material, similar to conventional LIBs.^[^ ^14^, ^15^, ^16^, ^17^, ^18^, ^19^, ^20^, ^21^ ^]^ Additionally, the absence of the reactive lithium metal in the pristine state offers practical benefits in terms of handling, cell assembly, production, and R&D experimentation efforts.

Nevertheless, ZELMBs are not applicable yet as the plating/stripping behavior of Li on Cu is inhomogeneous, inefficient, and suffers from intense active lithium loss (ALL) as schematically shown in Figure 1b.^[^ ^22^ ^]^ Given the absence of a Li reservoir via a Li foil (= LMB), the ALL cannot be compensated and results in pronounced capacity loss and fading (Figure 1c).

The design of the cell, current collector, and electrolyte can influence the performance and viability of ZELMBs,^[^ ^24^ ^]^ for example, via high‐concentration electrolytes,^[^ ^25^ ^]^ dual salt electrolytes,^[^ ^25^, ^26^ ^]^ electrolyte additives,^[^ ^27^ ^]^ or solid‐state electrolytes.^[^ ^28^ ^]^ While they aim to *decrease* ALL, the pre‐lithiation approach can be a complementary strategy to *compensate* them and even create a Li reservoir (Figure 1d). Though, ZELMBs exhibit rather a low technology readiness level, their beneficial handling and intense ALL render them a suitable system for prelithiation R&D.

**Figure 2:**
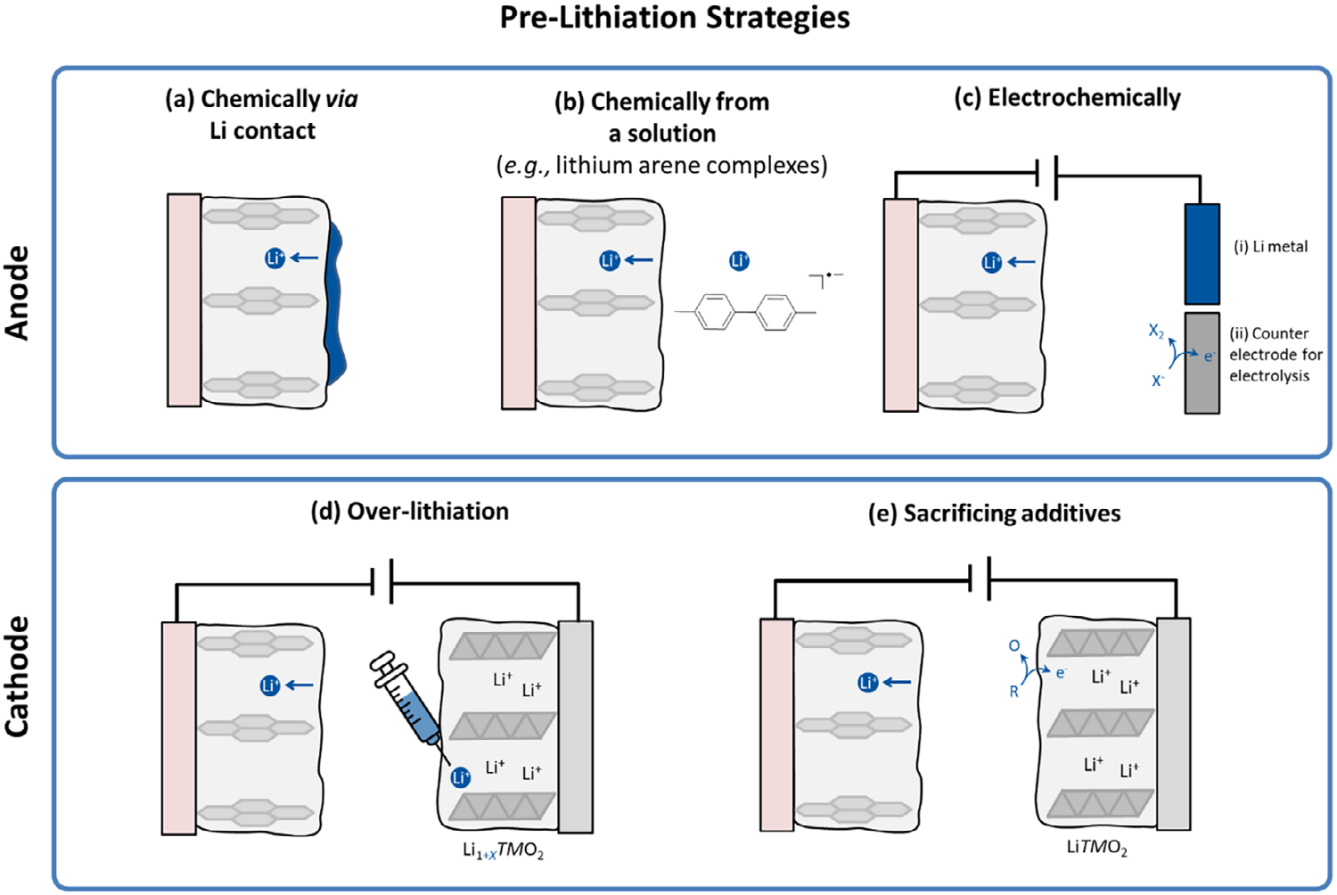
Overview of prelithiation strategies, which can be realized on *anode* a) chemically via a Li contact, b) from a solution via respective Li‐based chemicals, c) electrochemically within (i) a Li cell or via (ii) electrolysis. Prelithiation can also proceed on the cathode d) via overlithiation or e) via sacrificing additives. Redrawn from ref.[32]

As summarized in **Figure** **2**, the prelithiation conventionally proceeds directly on the anode, either during direct Li metal contact (Figure 2a), chemically via Li arene complexes (Figure 2b), or electrochemically, i.e., galvanostatically in a Li cell or during electrolysis (Figure 2c). Prelithiation can also proceed via the cathode by overlithiation, that is, by adding extra active Li in the Li layer of a respective cathode before cell assembly (Figure 2d). All these approaches require additional processing/assembly steps, an inert atmosphere because of reactive agents, and enhance the risk of material deterioration. More promising is the additive approach, where a sacrificing agent is added to purposely oxidize during the first charge and to provide active Li to the cell (Figure 2e). Lithium squarate (Li_2_C_4_O_4_) is known as a promising sacrificial additive^[^ ^29^ ^]^ oxidizing at a relative low voltage, but remains mechanistically ambiguous. For instance, decreasing the amount of Li_2_C_4_O_4_ within an NCM622 (LiNi_0.6_Co_0.2_Mn_0.2_O_2_) cathode increases the oxidation voltage from 4.3 to 4.7 V.^[^ ^29^ ^]^ With lithium iron phosphate (LFP) cathodes, the additive oxidation occurs at even lower cathode potentials, apparently due to the catalytic effects of charged LFP.^[^ ^30^ ^]^ This inconstancy underscores the importance of unravelling their working principles to optimize both the conditions and effectiveness.^[^ ^31^ ^]^ Also, as investigated in this work, the working principle fundamentally differs when incorporated via cathode versus electrolyte.

## 2 Results & Discussion

### 2.1 Pre‐Lithiation via Li_2_C_4_O_4_: Additive‐in‐Electrolyte Versus Additive‐in‐Cathode

The ZELMB cells consist of an NCM622‐based cathode with incorporated ≈1.25 ± 0.02 mg Li_2_C_4_O_4_ additive and a total mass loading of ≈10.8 ± 0.5 mg cm^−2^, further referred as “Additive‐in‐cathode”. In parallel, an equivalent amount of ≈1.26 ± 0.01 mg of Li_2_C_4_O_4_ is dispersed in an electrolyte composed of 0.8 M lithium difluoro(oxalato)borate (LiDFOB) in a solvent mixture of ethylene carbonate and ethyl methyl carbonate (EC:EMC 3:7, by wt.) and is further termed as “Additive‐in‐electrolyte”.

**Figure 3:**
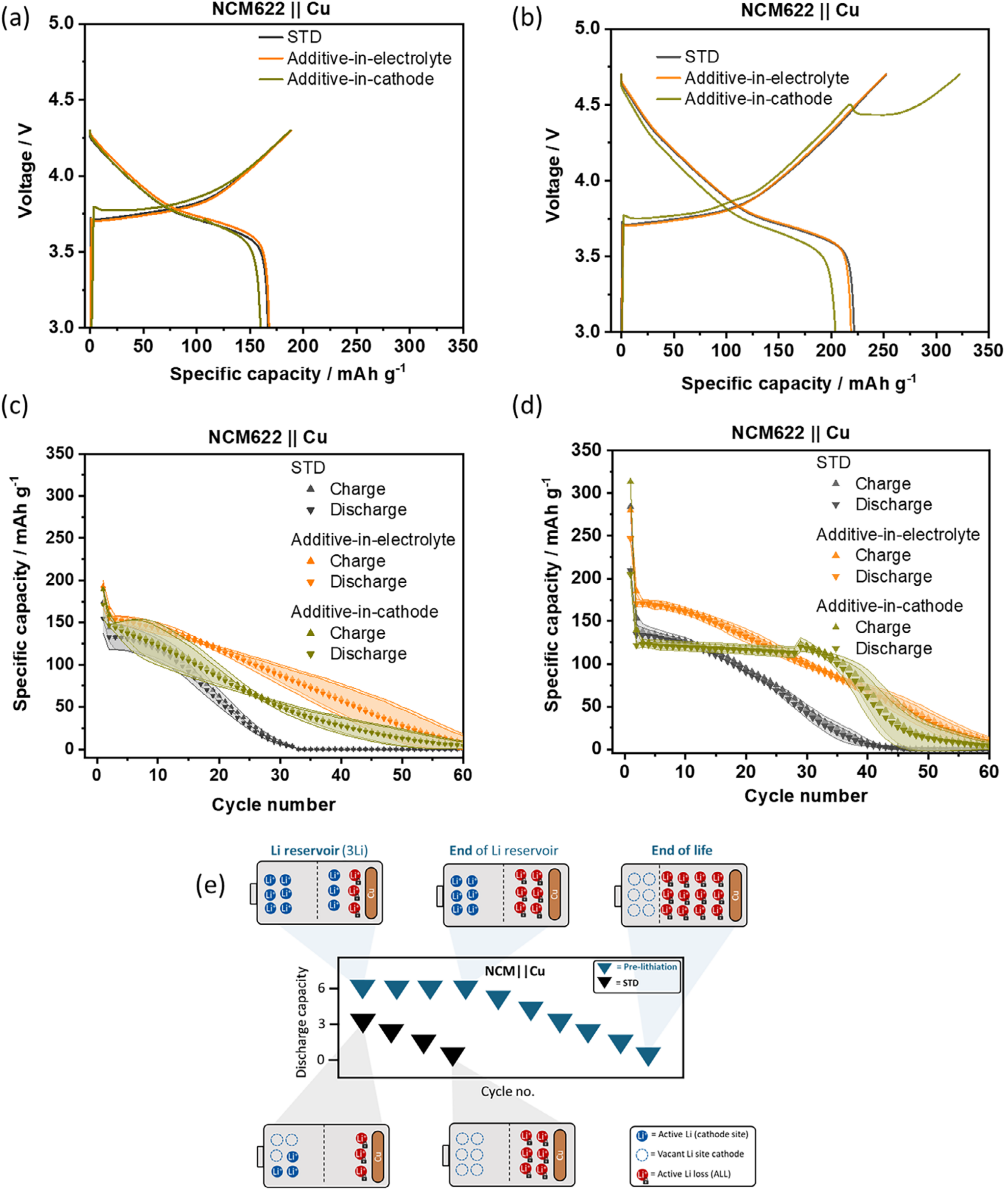
Electrochemical data of NCM622||Cu cells with STD, additive‐in‐electrolyte, and additive‐in‐cathode. Initial charge/discharge cycle in the voltage window a) 4.3–3 V and b) 4.7–3 V; at 0.1C (1C ≙ 180 mA g^−1^). Specific charge and discharge capacities as a function of cycle number within c) 4.3–3 V and d) 4.7‐3 V in first cycle (“additive activation”) followed by 4.3–3 V. e) In pre‐lithiated cells the Li inventory is high, i.e., the cathode is fully lithiated and ‐depending on pre‐lithiation degree–the anode has a Li reservoir (see Figure 1d). During ongoing charge/discharge cycling and ALL, the Li inventory decreases, and after depletion of the Li reservoir, the cells start to fade.

The charge/discharge cycling data for both approaches and additive‐free ZELMBs (= STD) are shown in **Figure** **3**. Though Li_2_C_4_O_4_ does not oxidize in cells with an upper cut‐off voltage (UCV) of 4.3 V as shown in Figure 3a, the additive slightly enhances the cycle life, as shown in Figure 3c, and can be related with SEI alteration, which in‐depth investigation is not the focus of *this* work. As illustrated in Figure 3b for a UCV of 4.7 V, an oxidation plateau is seen at ≈4.5 V, but only for the additive‐in‐cathode. Here, the initial specific charge capacity is 322.6 mAh g^−1^, thereby providing an additional specific capacity of 70 ± 5 mAh g^−1^ compared to STD which is even higher than the calculated value of 49.7 ± 1 mAh g^−1^ according to Faraday law and will be discussed later. Interestingly, additive‐in‐electrolyte does not show any oxidation during the initial charge (Figure 3b).

The cycle life, shown in Figure 3d, indicates improved capacity retention for the additive‐in‐cathode, before a “rollover” fading starts after cycle no. 30. This rather linear capacity retention can be attributed to the consumption of the created lithium reservoir in the course of prelithiation in the first cycle (Figure 3e). Though, no prelithiation observed of “additive‐in‐electrolyte”, the capacity retention is improved compared to STD and can be related with improved SEI similar to cycle life in the voltage range of 4.3–3.0 V (Figure 3c).

### 2.2 Effects of Li_2_C_4_O_4_ Additive on SEI and Gas Formation

**Figure 4:**
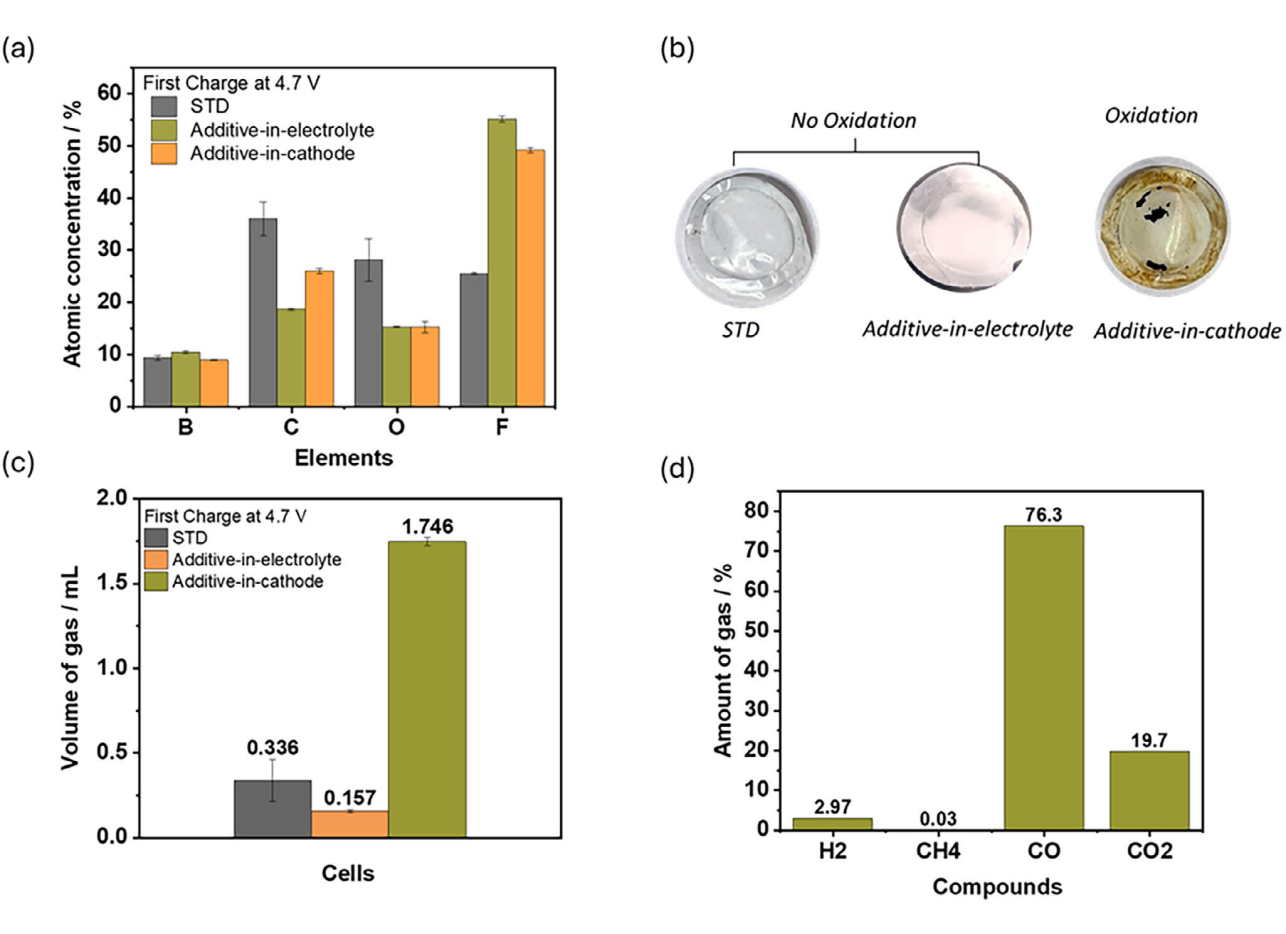
Post mortem investigation of NCM622||Cu pouch cells with STD, additive‐in‐electrolyte, and additive‐in‐cathode. a) EDX data of relative atomic concentrations of deposited layer on the Cu electrode after 60 cycles b) decomposition reactions on separators (cathode side) c) GC‐BID data of the relative amount of gas obtained after 20 cycles. d) Gas composition identified in cells with additive‐in‐cathode after 20 cycles.

The surface analysis after 60 charge/discharge cycles via energy‐dispersive X‐ray spectroscopy (EDX) is shown in **Figure** **4**a. Additive‐in‐electrolyte cells exhibit the highest presence of fluorinated compounds (55%) based on the atomic concentration of fluorine (F) compared to additive‐in‐cathode (48%) and STD cells (25%). According to literature, F‐based species can be beneficial for SEI,^[^ ^33^, ^34^ ^]^ and the enhanced F amount in additive‐in‐electrolyte can be related to the result from reactions of Li_2_C_4_O_4_ with LiDFOB. Additive‐in‐electrolyte cells also show lower atomic concentrations of carbon (18%) and oxygen (15%), compared to STD cells with 36% and 28% respectively, and may correlate with improved SEI and cycle life.

While intrinsic decomposition of Li_2_C_4_O_4_ is claimed to predominantly provide CO, the oxidative decomposition can theoretically proceed via two pathways, as shown by Equation 1,^[^ ^29^, ^30^ ^]^ and Equation 2,^[^ ^29^, ^30^, ^35^, ^36^ ^]^ where either carbon dioxide (CO_2_) or carbon monoxide (CO) is produced. Hence, these reactions can also occur simultaneously at respective conditions as reported with LFP‐based cells, where the FePO_4_ apparently acts as a catalyst.^[^ ^30^ ^]^

(1) $$Li_{2}C_{4}O_{4}\to 2CO_{2}+2C+2e^{-}+2Li^{+}\;$$

(2) $$Li_{2}C_{4}O_{4}\to 4CO+2e^{-}+2Li^{+}$$

Data from gas chromatography with barrier discharge ionization detection (GC‐BID) shown in Figure 4c, indicate highest amount of gas generation (1.746 mL) for additive‐in‐cathode cell and can be related to oxidation of Li_2_C_4_O_4_ in first cycle (Figure 3b), where 76% is CO (Equation 2) and 20% CO_2_ (Equation 1), as indicated in Figure 4d. Decomposition reactions can be also observed by residuals and color change of the separator as shown in Figure 4b. The STD cell produces 0.336 mL gas, where the LiDFOB is suggested to relevantly contribute to gas formation in the course of SEI formation.^[^ ^30^, ^37^, ^38^, ^39^ ^]^ Cells with additive‐in‐electrolyte have less gas (0.157 mL) and additionally hint to the involvement in SEI process, which minimizes the amount of LiDFOB reduction.

### 2.3 Influence of Li_2_C_4_O_4_ on Electrodes

**Figure 5:**
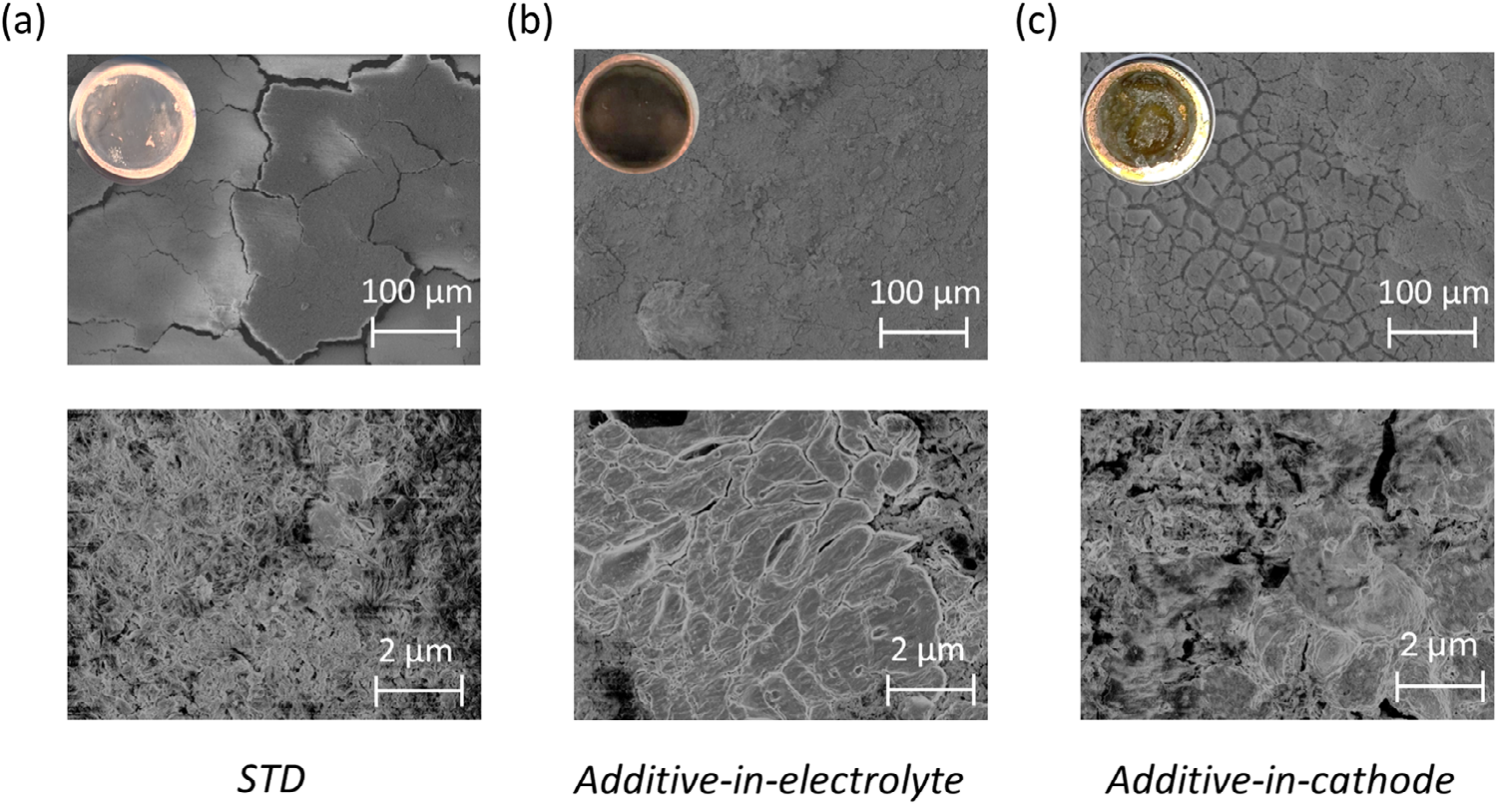
SEM images of the top view of Li‐depositions on Cu after 60^th^ cycle with first cycle charge voltage at 4.7 V, followed by cycling within 4.3–3 V from a) STD, b) additive‐in‐electrolyte, and c) additive‐in‐cathode. The additive tends to homogenize and densify the plated Li, thus relevantly impacts the anode, likely via SEI contributions.

Li plating and stripping on Cu is investigated via scanning electron microscopy (SEM) and shown in **Figure** **5** after 60 cycles in the discharged (stripped) state cycled with “additive‐activation” protocol, i.e., initial charge voltage at 4.7 V followed by charge/discharge cycling within 4.3–3 V. The STD cell displays inhomogeneities, characterized by HSAL and surface cracking (Figure 5a), and can be correlated with the observed capacity fade in Figure 3d. Additive‐in‐electrolyte cells show dense and homogeneous lithium plating as shown in Figure 5b. In contrast, the additive‐in‐cathode cells indicate a mixture of HSAL and dense lithium plating, as shown in Figure 5c. Both configurations with Li_2_C_4_O_4_ show an improved plating morphology compared to the STD cell, proving its relevant impact on the anode.

**Figure 6:**
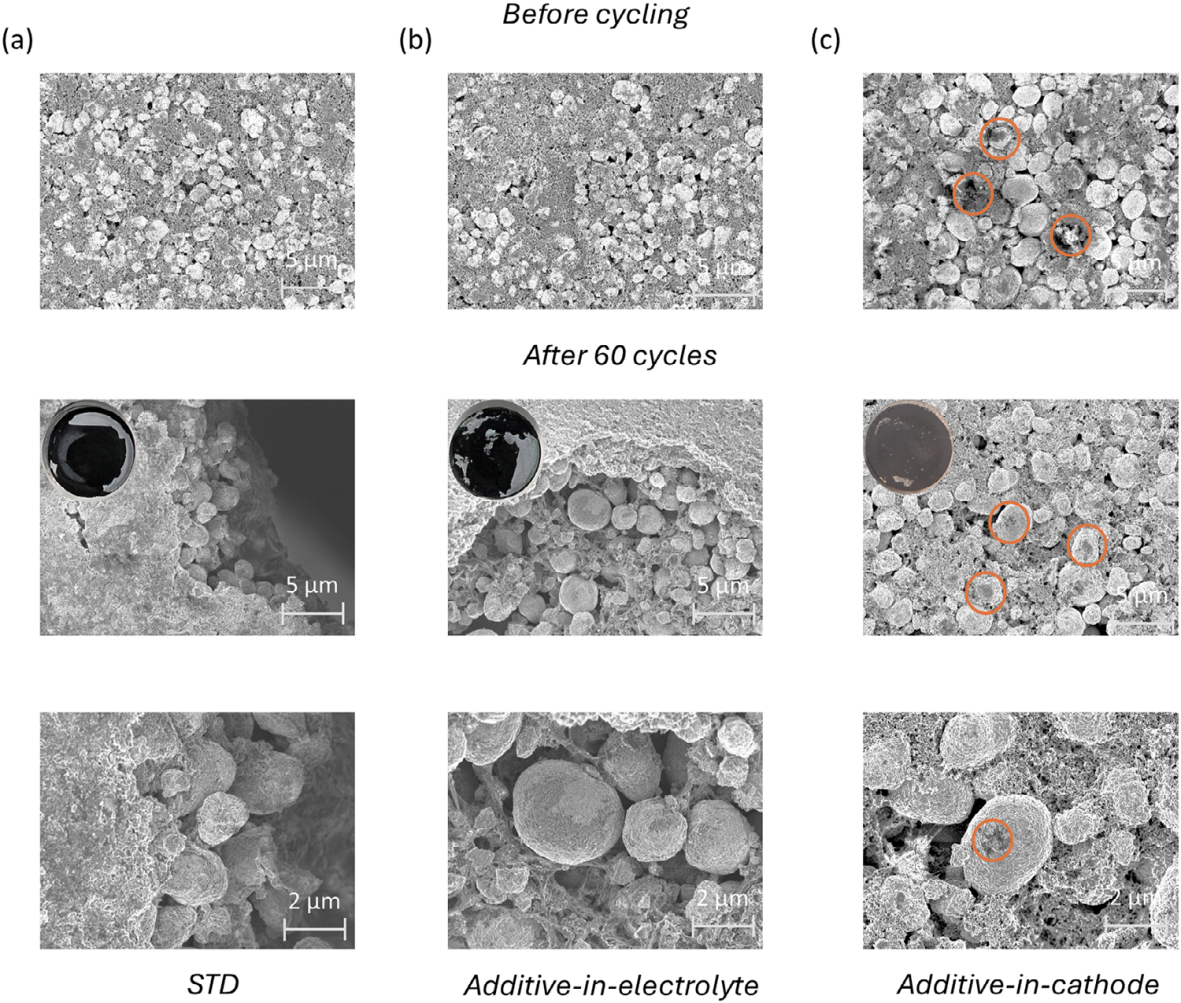
Top‐view SEM images of NCM622 cathode before and after 60 cycles of a) STD, b) Additive‐in‐electrolyte, and c) Additive‐in‐cathode, showing the presence and residuals of additive before and after cycling (orange cycles), respectively, as well as porosity increase after cycling (electrode inset, 2^nd^ raw).

The SEM images of NCM622 electrodes before and after cycling are shown in **Figure** **6**. The NCM622 electrode for STD and additive‐in‐electrolyte cells is intact and shown in Figure 6a,b. Additive‐in‐cathode (Figure 6c) indicates dark spots before cycling, representing Li_2_C_4_O_4_ additives, which disappear after cycling in the course of oxidation (Figure 3b) and generate voids as shown in Figure 6c due to gas generation (Figure 4c). The higher porosity is disadvantageous as it decreases the density and likely deteriorates the composite network and kinetics.^[^ ^40^ ^]^

**Figure 7:**
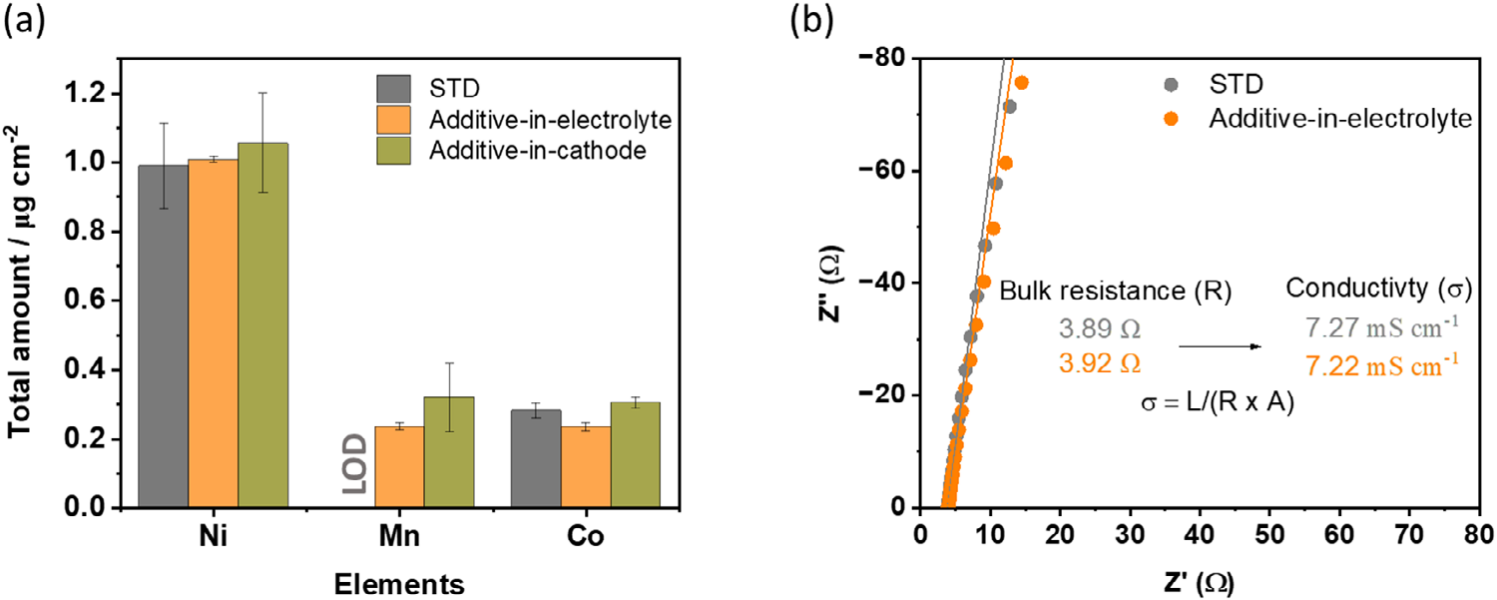
a) *TM* amount on Cu electrodes after 60 cycles by means of ICP‐OES. b) Resistance obtained via EIS indicating a similar ionic conductivity of 7.2 mS m^−1^ in STD and additive‐in‐electrolyte cells.

Inductively coupled plasma optical emission spectroscopy (ICP‐OES) indicates similar amounts of Nickel (Ni), Cobalt (Co), and Manganese (Mn) after 60 cycles (**Figure** **7**a) and excludes the impact of transition metal (*TM*) dissolution and electrode crosstalk.^[^ ^41^, ^42^, ^43^ ^]^ Also, the electrolyte resistance/ionic conductivity of STD and additive‐in‐electrolyte is similar (7.2 mS m^−1^) as concluded from electrochemical impedance spectroscopy (EIS), shown in Figure 7b, and can be excluded to have a relevant impact on charge/discharge cycling.

### 2.4 Oxidation Behavior of Li_2_C_4_O_4_ During Overcharge Experiments

**Figure 8:**
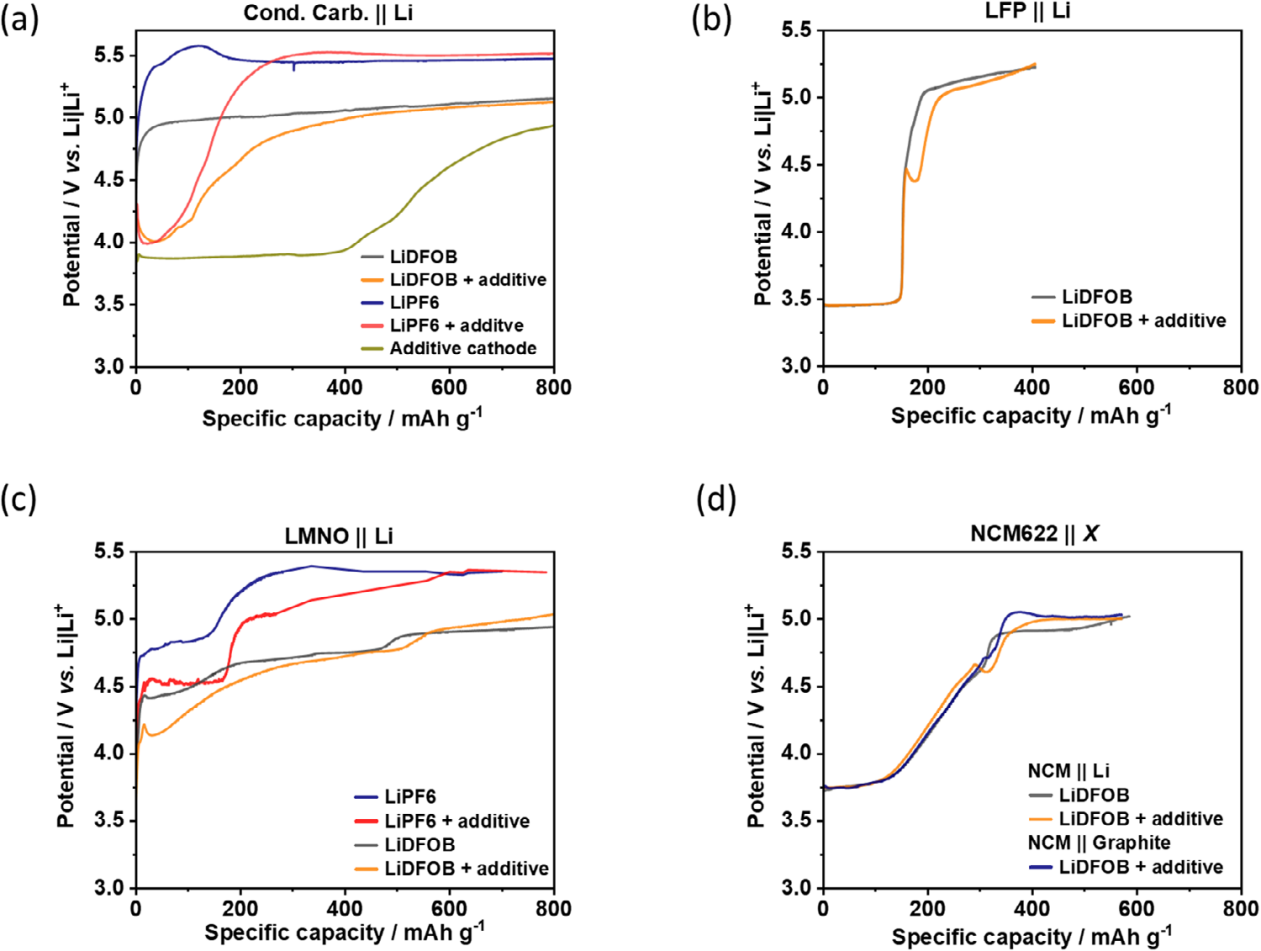
Overcharge experiments within different cell setups and electrolytes at 0.1C: a) Cond. Carbon||Li, b) LFP||Li c) LNMO||Li and d) NCM622||Li vs NCM622||Graphite. The oxidation onset of squarates depends on the cathode type. Its presence in general depends on the anode, i.e., whether the anode consumes the additive in course of SEI formation.

The anodic stability is thoroughly investigated via overcharge experiments on different cathode types according to the literature.^[^ ^44^, ^45^, ^46^ ^]^ As previously reported, the anodic bulk stability of conventional LiPF_6_/carbonate‐based electrolytes is ≈5.4 V vs Li|Li^+^ as shown in **Figure** **8**a on conductive carbon (Cond. carb.)‐based composite electrode (= active material‐/*TM*‐free). With the squarate additive, the electrolyte oxidation starts at ≈4.0 V vs Li|Li^+^ and, after additive consumption, reaches the bulk oxidation plateau of 5.4 V vs Li|Li^+^ again. Replacement of the LiPF_6_ with LiDFOB (= STD in this manuscript) decreases the electrolyte oxidation plateau to ≈5.0 V vs Li|Li^+^ while the additive‐containing electrolyte remains at ≈4.0 V vs Li|Li^+^. Hence, the LiDFOB is oxidatively less stable than LiPF_6_^[^ ^47^ ^]^ and the squarate starts to oxidize at low cathode potential independent of the conducting Li salt. When directly incorporated in the cathode, the squarate oxidizes at even lower cathode potential (≈3.8 V vs Li|Li^+^).

As expected, given the delithiation of LFP at lower cathode potentials (≈3.7 V vs Li|Li^+^), the LFP||Li cells have no indications for electrolyte oxidation in the LFP delithiation range (Figure 8b), which would be seen via charge capacity shifts to higher values and/or additional plateaus.^[^ ^48^, ^49^ ^]^ However, contrary to conductive carbon electrodes the squarate oxidation starts at a higher cathode potential (≈4.4 V vs Li|Li^+^), while it also decreases the cathode potential afterwards to a local minimum (“spike”).

Overcharge experiments on LiNi_0.5_Mn_1.5_O_4_ (LNMO)‐based cathodes are shown in Figure 7c. The conventional LNMO delithiation proceeds via two potential plateaus within ≈4.7–4.9 V vs Li|Li^+^ with LiPF_6_‐based electrolyte. With STD, the LNMO delithiation interferes with LiDFOB oxidation, as seen by the absence of LNMO‐characteristic plateaus and a decreased oxidation onset potential to 4.4 V vs Li|Li^+^. With additive, this onset is further decreased to 4.2 V vs Li|Li^+^ and to 4.5 V vs Li|Li^+^ with LiPF_6_.

As shown in Figure 8d, the STD electrolyte in NCM622||Li cells only starts to oxidize after an almost full delithiation at ≈4.7 V vs Li|Li^+^, i.e., no indications for electrolyte oxidation are observed below this potential contrary to LNMO||Li cells in Figure 8c. With the squarate additive, electrolyte oxidation can be seen via a “spike”, similar as in LFP||Li cells in Figure 8b, but at a higher cathode potential of ≈4.7 V vs Li|Li^+^. Consequently, the *onset* of squarate oxidation sensitively depends on cathode type.

Interestingly, the oxidation phenomenon in general and its oxidation capacity depend on the anode type, as well. When the anode forms an SEI *in operando* during charge like on Cu in ZELMBs or on graphite in LIBs, it consumes electrolyte amount in the course of electrolyte reduction and likely depletes the additive (Figure 10), which correlates with its contribution to SEI formation as previously discussed. Additive absence correlates with the respective absence of oxidation plateau, as for example shown in NCM622||graphite cells in Figure 8d. In contrast, LMBs with an already passivated Li foil have less electrolyte reduction and consumption in the initial charge, and the additive amount is suggested to be higher and/or to remain nearly constant. Systematically higher amounts of squarate additives can be consequently suggested in LMBs, which is indicated by the respective oxidation plateau.

**Figure 9:**
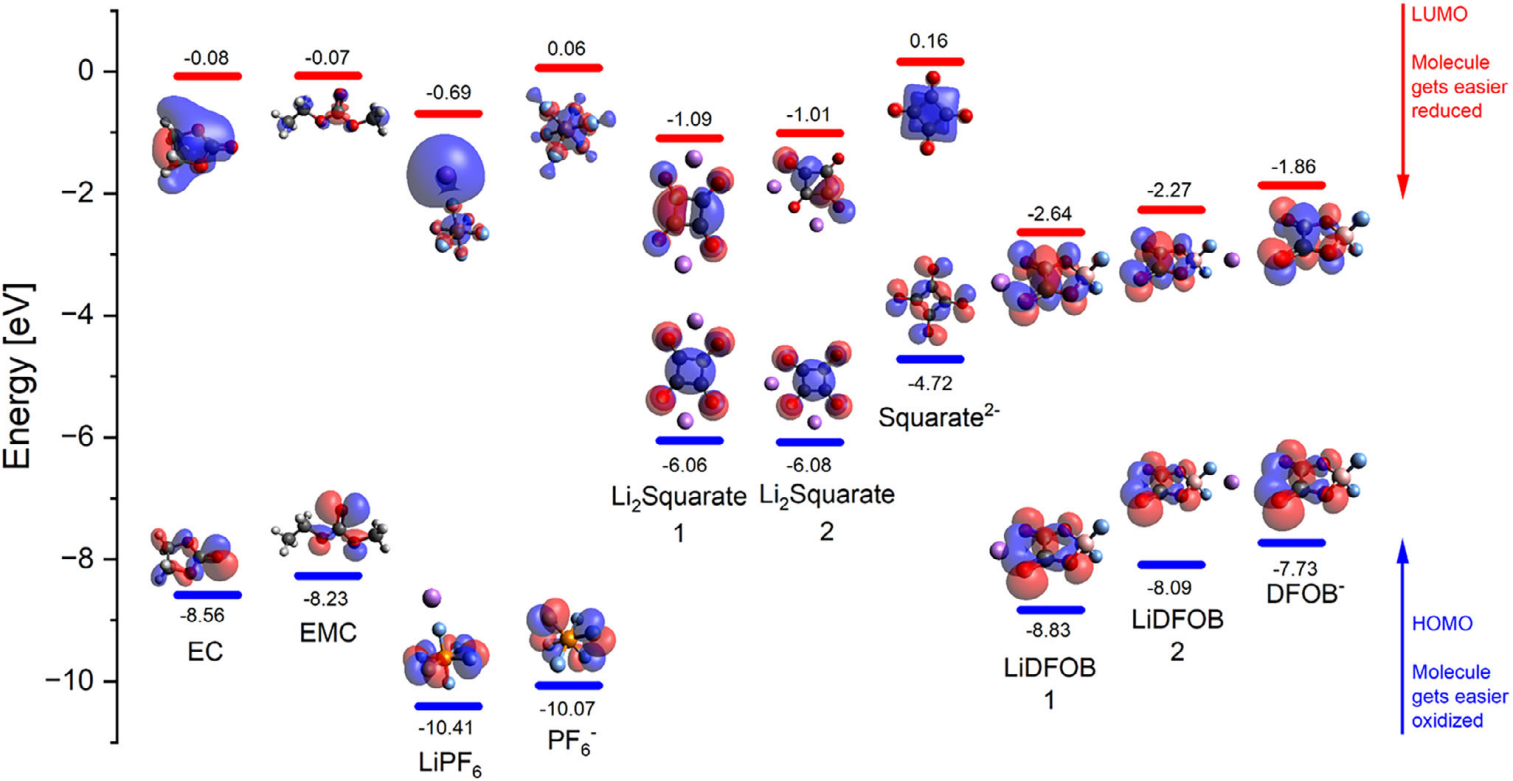
HOMO and LUMO energy values according to DFT calculations of solvents (EC and EMC), salts (LiPF_6_ and LiDFOB), lithium squarate (Li_2_C_4_O_4_), and single anions.

Squarate consumption via SEI formation suggests relative high reduction potentials, i.e., relatively low energetic level for the lowest unoccupied molecular orbital (LUMO) compared to the other electrolyte components. Indeed, via computational analysis using density functional theory (DFT) shown in **Figure** **9**, the LUMO is calculated to be −1.09 eV, which is lower than the solvents, though higher than LiDFOB (−2.64 eV). Consequently, its participation in reductive decomposition reactions, SEI formation and consumption can be concluded to be very likely in competition with LiDFOB, as previously discussed (see, e.g., Figure 4a).

When not consumed during reduction on anode (e.g., in LMBs) it readily oxidizes, as implied by the highest value of the highest occupied molecular orbital (HOMO) of the electrolyte components. This fits with the experimentation, where it oxidizes at relative low cathode potentials, i.e., before the other components. Also, a higher HOMO of DFOB^−^ than PF_6_^−^ is in line with the experiments where the DFOB^−^ oxidizes at lower electrode potentials than PF_6_^−^.

## 3 Conclusion and Future Perspectives

Among several prelithiation techniques, the approach via *a* sacrificing additive is promising for reasons of simplicity, costs, and scalability. Zero excess lithium metal battery (ZELMB) suffers from high active lithium loss (ALL) but is easy in handling and preparation, thus suitable for additive‐based prelithiation R&D.

**Figure 10:**
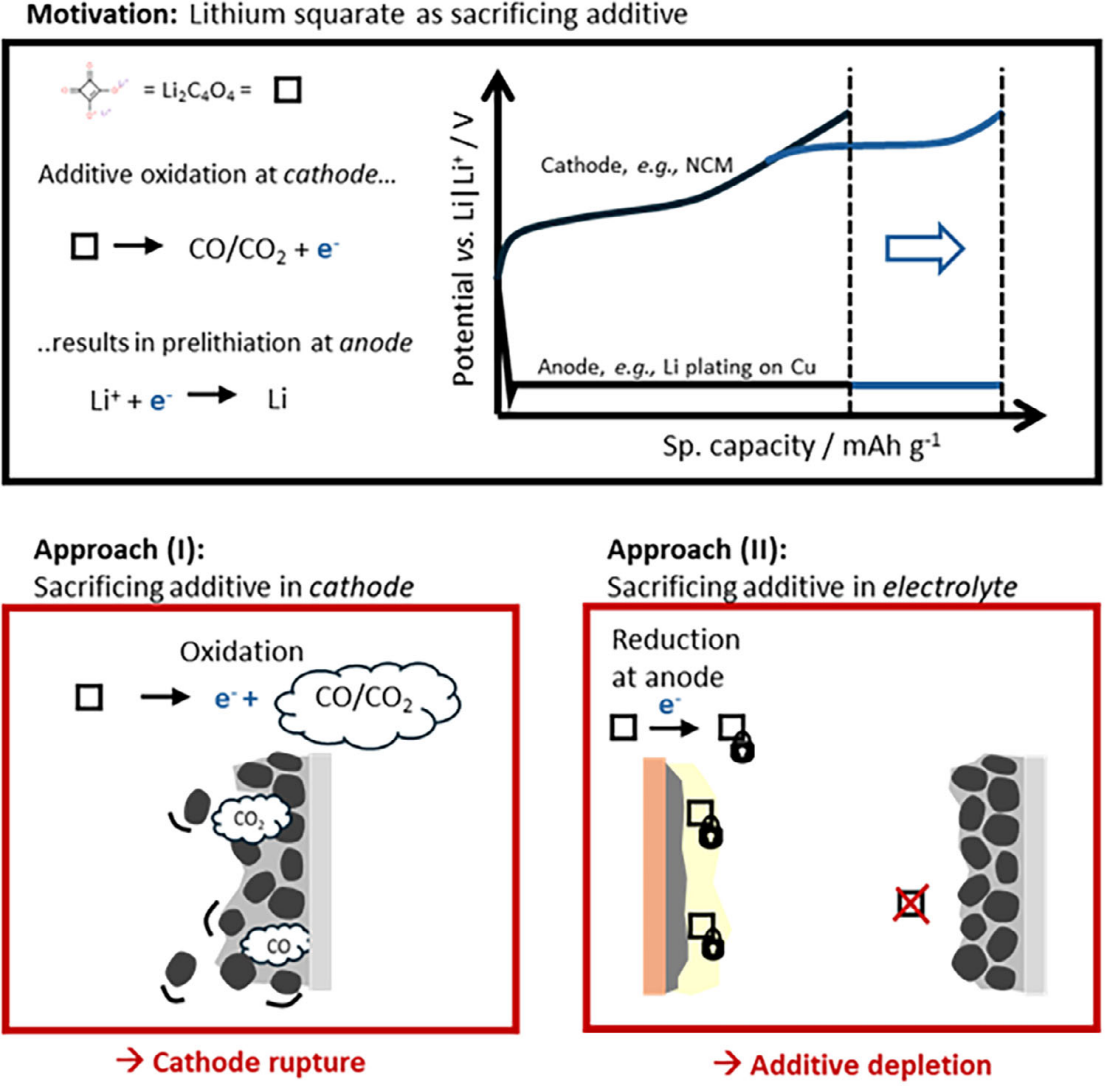
Schematic overview of prelithiation via sacrificing additives, here lithium squarate, which provides electrons, thus active Li in the course of the oxidation reaction. When applied within the cathode, this additive ruptures the cathode composite in the course of gas evolution. When applied via *the* electrolyte, the additive gets reduced, consequently consumed, and depleted.

Lithium squarate (Li_2_C_4_O_4_), a literature‐known sacrificing additive, is shown to oxidize at ≈4.7 V vs Li|Li^+^ in LiNi*_x_* Co*_y_* Mn*_z_* O_2_ (NCM)||Cu cells, when incorporated within the cathode composite. However, the application is limited due to enhanced porosity and pinholes in the course of gas generation, predominantly CO and CO_2,_ as shown by means of gas chromatography (**Figure** **10**).

In contrast, incorporating a similar additive amount via electrolytes does not pre‐lithiate this ZELMB setup and is attributed to its consumption in the course of solid electrolyte interphase (SEI) formation during initial charge and is also validated in NCM||graphite cells (Figure 10). According to computational analysis using density functional theory, the lowest unoccupied molecular orbital (LUMO) of Li squarate is indeed relatively low, suggesting a relative high reduction potential, readiness to react with freshly deposited Li, and correlates with contributions to SEI chemistry, as seen via energy dispersive x‐ray‐ and gas analysis, suppressed gas evolution, and more homogeneous plating on Cu. In Li metal batteries, where a native SEI already exists before cell charging, the squarate is less reductively consumed, consequently, an increased presence can be concluded. In fact, its oxidation is observed, and proves the principle.

Interestingly, the exact oxidation onset of this additive sensitively depends on the combination of conducting Li salt and cathode active material, as shown by overcharge experiments. The oxidation is characterized by a decrease in electrode potential and is speculated to cause additional delithiation capacity from the cathode for a fixed upper cut‐off voltage, thus be likely the cause for the observed higher additional charge capacity than theoretically calculated for the additive according to Faraday laws. Also, the presence of carbon ‐ an oxidation product from squarate ‐ can theoretically additionally oxidize and increase the respective charge capacity beyond the theoretically calculated value.

According to these findings, it can be concluded, that prelithiation via sacrificing additives in electrolytes is only possible when not reductively consumed during initial charge. However, it is aimed in systems with exactly those electrodes (e.g., Si), which have high ALL due to SEI formation. Furthermore, the high oxidation onset of Li squarate can be a challenge, as the conventional NCM has high Li^+^ extraction ratios at these potentials and can structurally deteriorate.

## 4 Experimental Section

### Lithium Squarate (Li_2_C_4_O_4_) Synthesis

Lithium Squarate (Li_2_C_4_O_4_) is prepared from 3,4‐dihydroxy‐3‐cyclobutene‐1,2‐dione (Sigma Aldrich, 99.0% purity) and lithium carbonate (Acros Organics, 99.0% purity), which were dissolved in 150 mL of deionized water at a 1:1 molar mixture. The solvent (deionized water) is evaporated gradually under reduced pressure at 40 °C with a Büchi rotary evaporator, and a fine powder of Li_2_C_4_O_4_ additive was obtained.

### Electrode and Electrolyte Preparation

The positive electrode was prepared by dissolving 95% active material of NCM622 with >98% purity (Merck Germany), 3% polyvinylidene difluoride (PVdF) binder (Solef 5130, Solvay), and 2% conductive carbon Super P (Imerys France) in N‐methyl‐2‐pyrrolidone NMP solvent (Sigma–Aldrich Germany) and mixed using a propeller dispermat at 2000 RPM for 1 h. The slurry made of 50% solid content is casted on an aluminum current collector by using an automatic film applicator and a doctor blade. The coated electrode sheets were dried in an oven at 80 °C for 2 h followed by calendaring to 50 % porosity and punched. Finally, the punched discs were vacuum dried in a Büchi B‐585 glass drying oven under reduced pressure (<5×10^−2^ bar) at 120 °C for 12 h. A mass loading of ≈11.5 ± 0.3 mg cm^−2^ was achieved. Cathode with Li_2_C_4_O_4_ additive is obtained by the same process with only by using only 85% NCM622 active material and 10% Li_2_C_4_O_4_ additive in the solid content, hence a mass loading of ≈10.8 ± 0.5 mg cm^−2^ was achieved.

1 M electrolyte was prepared by dissolving Lithium difluoro(oxalato)borate LiDFOB in EC: EMC 3:7 wt.% (E‐Lyte Innovations Germany). Electrolytes with Li_2_C_4_O_4_ additive are made by dispersing the additive in 0.8 M LiDFOB. The cells were assembled in a two‐electrode CR2032 coin cell setup using a Ø14 mm cathode, Ø15 mm copper current collector, and Ø16 mm Celgard 2500 separator with 2 spacers of 1 mm for all cells. 50 µL of electrolyte was used for each cell. The first cell setup was NCM622 |LiDFOB| Cu (STD) while the second was NCM622 |LiDFOB + Li_2_C_4_O_4_| Cu (Additive‐in‐ electrolyte) and the third cell made of NCM622 + Li_2_C_4_O_4_ |LiDFOB| Cu (Additive‐in ‐cathode).

### Electrochemical Investigation

The electrochemical investigation used a Maccor Series 4000 battery cell test system at 20 °C for all three cell setups; STD: NCM622 |LiDFOB| Cu, Additive‐in‐electrolyte: NCM622 |LiDFOB + Li_2_C_4_O_4_| Cu, and Additive‐in‐cathode: NCM622 + Li_2_C_4_O_4_ |LiDFOB| Cu. The test of the first cycle was carried out after 6 h at open‐circuit‐voltage (OCV) via constant current charging (CCC) at 0.1C to different first cycle upper cut‐off voltages of 4.3 and 4.7 V followed by constant current discharge (CCD) at 0.1C to 3.0 V followed by a loop of 60 cycles containing: CCC at 0.5C and CCD at 0.5C with a 5 mins final rest step.

### Gas Chromatography with Barrier Discharge Ionization Detection(GC‐BID)

Pouch cells made of 5 x 4 cathode (NCM622) and 5.4 x 4.4 anode (Cu) were made for GC‐MS measurement. GC‐MS measurements were done using a GC‐2010 Plus system. The GC system was equipped with a PLOT gas separation column RT‐Msieve 5A (30 m x 0.32 mm x 30 µm) and a packed RT‐ShinCarbon ST (80/100, 2.0 m x 0.53 mm, both Restek GmbH, Bad Homburg, Germany). For the quantification, a BID‐2010 Plus and a TCD‐2010 Plus detector were used (all Shimadzu Deutschland GmbH, Duisburg, Germany). Manual injection of the samples was performed. Parameters were set according to the previously published method.^[^ ^50^ ^]^

### Scanning Electron Microscope (SEM)

An SEM instrument made from Carl Zeiss AURIGA; *Carl Zeiss Microscopy GmbH* was used for morphology characterization of the Cu and NCM 622 electrodes. SEM images were taken using a working distance of 5 mm and an accelerating voltage of 3 kV at different magnifications. To investigate the elemental composition on the Cu electrode, energy‐dispersive X‐ray spectroscopy (EDX) was used. The measurement was done with an Ultim Extreme detector. The spectra were evaluated with the INCA software (*Oxford Instruments*).

### Microwave Digestion

The microwave digestions were performed using a Multiwave 7000 (AntonPaar, Austria). After the disassembling of the cells in a glove box under argon atmosphere, the negative electrodes were rinsed with ≈1 mL DMC, respectively, and dried within the glove box to avoid reactions with water and oxygen. The samples were further digested in 2 mL deionized Water, 3 mL nitric acid, and 1 mL hydrochloric acid. The temperature program of the microwave consisted of a linear temperature gradient from 40 to 260 °C over a period of 12 min, following a steady temperature plateau and a pressure of 110 bar for 30 min. Afterward, the samples were diluted to 30 mL using deionized water (18.2 MΩ cm‐2, 5 ppb TOC, Merck Millipore, Germany).

### Inductively Coupled Plasma Optical Emission spectroscopy (ICP‐OES)

The quantification of TM´s on the negative electrode was performed using an SPECTRO ARCOS ICP‐OES (Spectro Analytical Instruments GmbH, Germany) equipped with a Scott spray chamber, a cross‐flow nebulizer, and an axial positioned plasma torch. A plasma power of 1400 W was applied, while a coolant flow of 12.00 l min^−1^, an auxiliary flow of 1.00 l min^−1^, and a nebulizer flow of 0.90 l min^−1^ were used. Instrumental control and data analysis were performed using the Smart Analyzer Vision (Spectro Analytical Instruments GmbH, Germany) software.

An external calibration using ICP calibration standards (LabKings B.V., Netherlands) was performed for all investigated elements. All samples and standards were diluted using 2 wt% nitric acid for nebulization optimization. The following emission lines were observed for the investigated elements during analysis: Li(670.780; 460.289 nm), Ni(231.604; 221.648; 341.476 nm), Mn(257.611; 259.373; 260.569 nm), Co(228.616; 238.892; 237.862 nm). Only one respective emission line was further analyzed, while the additional lines served as quality control. The method and further parameters were adapted from Evertz et al.^[^ ^51^ ^]^ and Vortmann et al.^[^ ^52^ ^]^

### Density Functional Theory Calculations

Calculations were performed in the framework of a density functional theory (DFT) computational method^[^ ^53^, ^54^ ^]^ as implemented in the ORCA package.^[^ ^55^, ^56^, ^57^ ^]^ Frontier orbital energies were calculated using the 6‐311++G** Pople‐style basis set, with double polarization functions to enhance the flexibility and with double diffuse functions to improve the calculation of species with extended electronic densities such as anions. The B3LYP functional^[^ ^58^, ^59^, ^60^, ^61^ ^]^ was used for the description of the exchange‐correlation potential. To account for the solvation, the conductor‐like continuum polarization model (C‐PCM)^[^ ^62^ ^]^ was used, and all molecules were simulated in ethylene carbonate (ε = 90.5^[^ ^63^ ^]^). All systems were optimized with respect to their geometry before any energy calculation was performed.

## Conflict of Interest

The authors declare no conflict of interest.

## Data Availability

The data that support the findings of this study are available from the corresponding author upon reasonable request.
